# Lower urinary tract symptoms in an elderly women caused by degeneration of the pubic symphysis

**DOI:** 10.1186/s12894-022-01052-1

**Published:** 2022-07-06

**Authors:** Kaixing He, Jinguo Wang, Haixiao Zhao, Jialin Gao

**Affiliations:** Department of Urology, The First Affiliated Hospital of Jilin University, No 71 Xinmin Street, Changchun, 130000 Jilin Province People’s Republic of China

**Keywords:** Lower urinary tract symptoms, Bladder, Pubic symphysis, Degenerative changes, Case report

## Abstract

**Background:**

Lower urinary tract symptoms are very common in elderly women, and transvaginal delivery and multiple deliveries have been confirmed to be risk factors. Transvaginal delivery and multiple deliveries may lead to an increase in pubic symphysis degeneration.

**Case presentation:**

A 79-year-old woman consulted a urologist because of worsening lower urinary tract symptoms such as frequent urination and urodynia. Color ultrasound and cystoscopy suggested the possibility of a bladder mass. A lump on the anterior wall of the bladder was observed although the surface mucosa was normal. Physical examination showed obvious tenderness in the posterior area of the pubic symphysis. Further urological computed tomography (CT) and pelvic magnetic resonance imaging (MRI) showed a nodular bony protuberance in the posterior part of the pubic symphysis, which was more obvious than before, with compression changes near the anterior wall of bladder. Open pelvic surgery showed that nodular bone tissue originating from the pubic symphysis significantly oppressed the anterior wall of the bladder behind the pubic symphysis. After resection of the nodule, the lower urinary tract symptoms were relieved significantly.

**Conclusions:**

Pubic symphysis degeneration caused by transvaginal delivery may be an important cause of lower urinary tract symptoms in women. Pelvic CT or MRI is necessary to diagnosis this condition.

**Supplementary Information:**

The online version contains supplementary material available at 10.1186/s12894-022-01052-1.

## Background

Lower urinary tract symptoms are important factors that affect the quality-of-life of elderly women. Urinary tract infections, an overactive bladder, and relaxation of pelvic floor muscles are common causes of lower urinary tract symptoms [1]. However, in recent years, several cases have reported abnormal changes after pubic symphysis that led to lower urinary tract symptoms in elderly women, such as a pubic symphysis cartilage cyst, abscess, or osteochondritis. The diagnosis and treatment was complicated due to a lack of understanding of lower urinary tract symptoms caused by abnormal changes after pubic symphysis. We report a case of lower urinary tract symptoms in an elderly woman caused by retropubic symphysis degeneration, which we consider may be helpful for making the correct diagnosis and treatment strategies.

## Case presentation

**Fig. 1:**
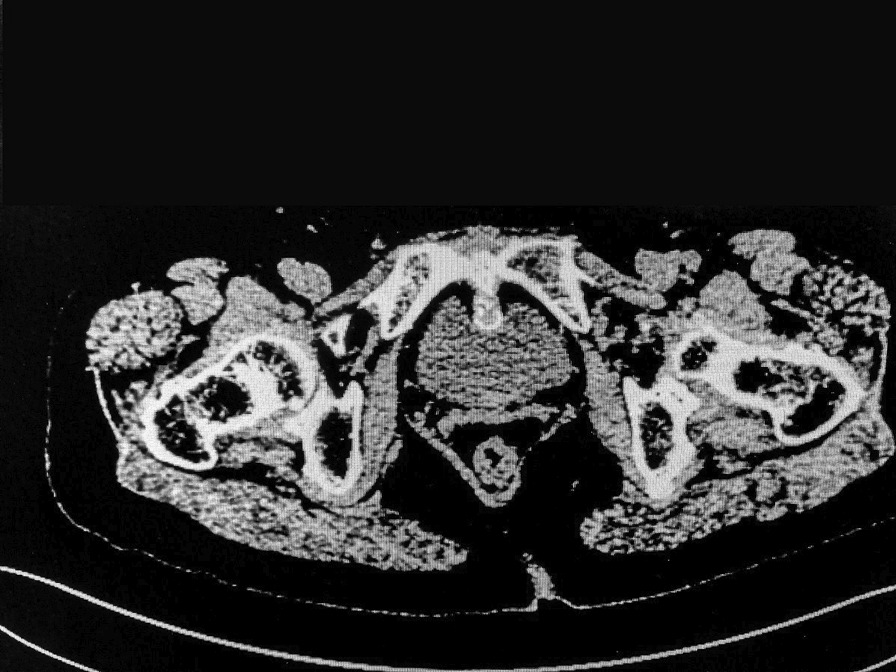
CT showing abnormal changes at the back of the pubic symphysis and compression of the adjacent anterior bladder wall

**Fig. 2:**
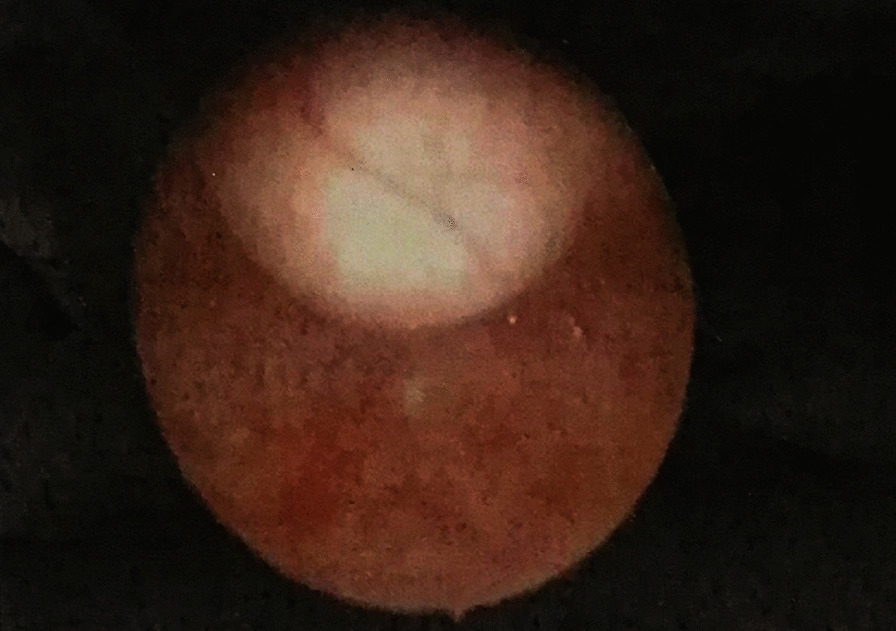
Cystoscopy showing that the mucosa of the bladder triangle and posterior wall was ruddy, with a bulge of about 2.0 × 1.5 cm in size on the top wall. The surface mucosa is normal, which appears to be caused by extravesical compression

Two years ago the patient, a 79-year-old woman, had complained of frequent urination and urodynia for which there was no obvious cause. The painful urination resulted in continuous severe pain behind the pubic bone after urination, forcing the patient to tilt forward with flexion and not dare to straighten-up. The pain was relieved gradually after about 15 min, but reoccurred at the next urination. Bladder color ultrasound revealed a bladder mass, but there was no gross hematuria. One year ago, the patient was treated in our hospital, with a urinary CT and pelvic MRI identifying an anterior calcified bladder nodule, adjacent to the pubic symphysis. The nodule was considered to be a lesion occupying the pelvic space, although gynecological examination showed no abnormality. Accordingly, the patient was referred to the orthopedic department for further treatment. However, due to the close proximity of the nodule with the bladder and its special anatomical position, the patient was not treated in the orthopedic department, and was requested to attend regular rechecks. Because the above symptoms became aggravated the patient attended our hospital for further treatment. Bimanual diagnosis: Tenderness in the posterior area of the pubic symphysis was obvious. A pelvic separation test was negative. Reexamination of the pelvic CT and a cystoscopy showed nodular bony processes in the posterior part of the pubic symphysis, which were more obvious than before, with compression of the adjacent anterior wall of the bladder. However, no clear foreign body was observed in the bladder cavity and the mucosa of the protuberant part of the bladder was normal (Figs. 1, 2). The patient was asked about her medical history and described no history of either transurethral foreign body implantation, pelvic surgery, or trauma. She described a history of tuberculosis, although current examinations showed no evidence of the disease. The patient had complained of osteoporosis for 10 years and had received bone peptide injections every year. No abnormality was found in her calcium and phosphorus metabolism and serum PTH level. An orthopedic consultation was arranged to consider the possibility of hyperosteogeny.

**Fig. 3:**
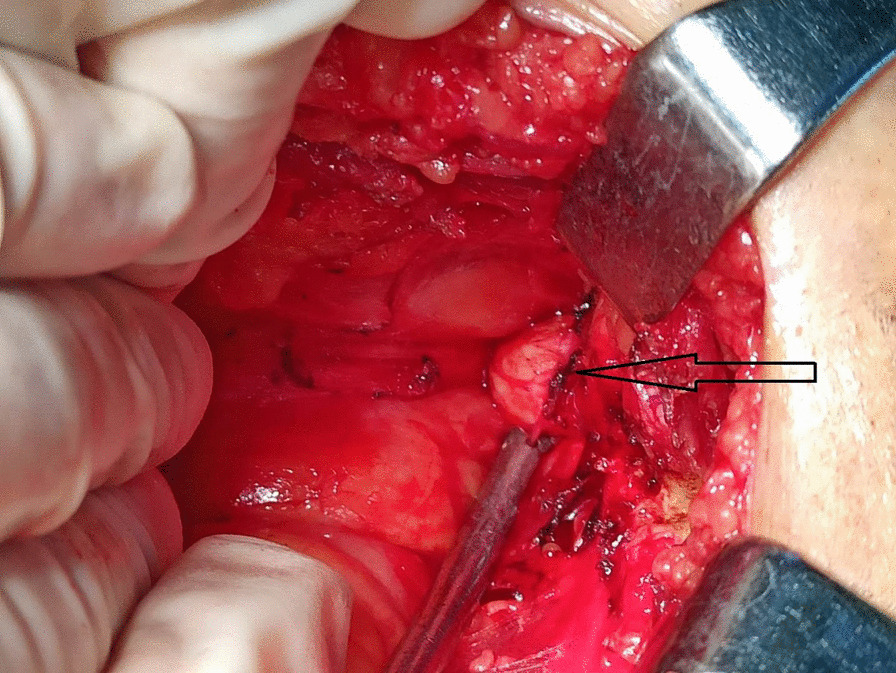
Nodular and hard bone tissue from the pubic symphysis seen behind the pubic symphysis, with a size of about 1.5 cm. The surface is round and blunt, while the boundary with the bladder serosa layer is not clear. (Shown at the arrow)

**Fig. 4:**
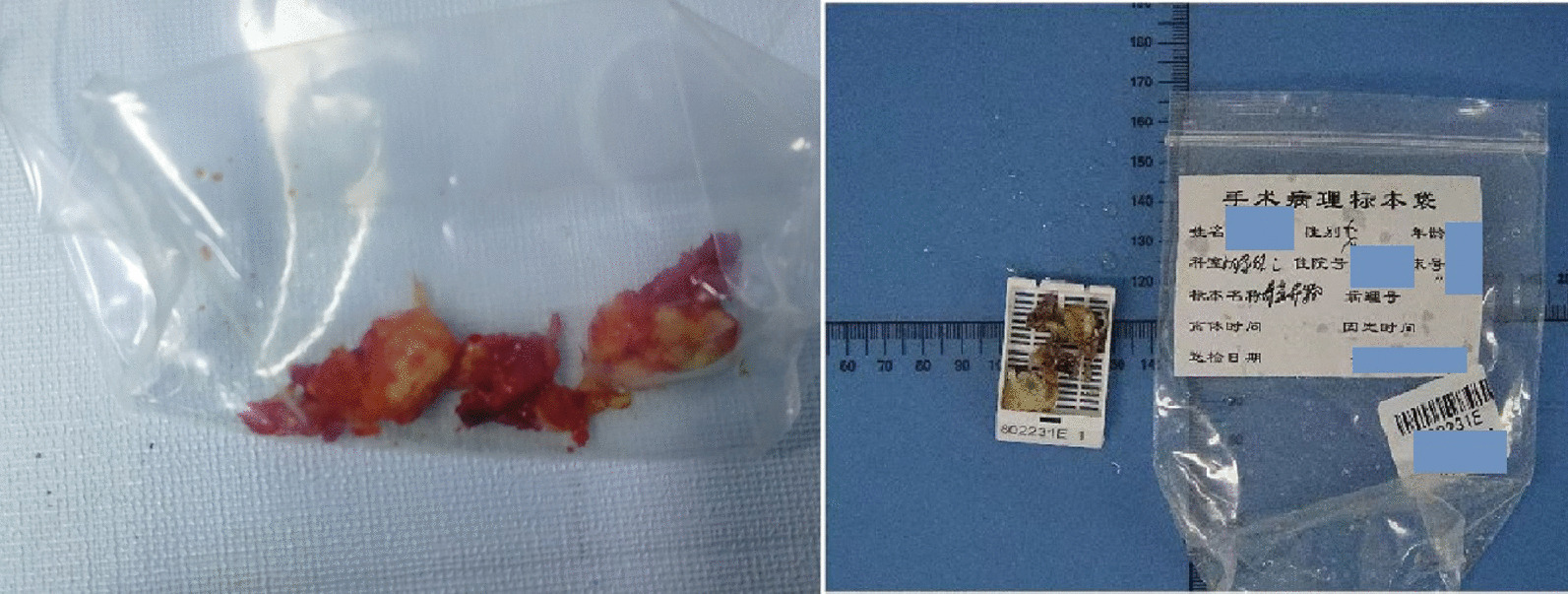
Pathological specimen: The texture of the nodule is white and slightly soft compared with the bone. Pathological report: Degenerative cartilage tissue, in which radial fine needle like crystals are seen that have destroyed the cartilage tissue with formation of local calcification. These changes may be caused by urate deposition

We finally decided to perform open pelvic surgery. During the operation, nodular bone tissue from the pubic symphysis was seen to be obviously oppressing the anterior wall of the bladder, surrounded by inflammatory changes that adhered to the serosa layer of the bladder with no clear boundaries (Fig. 3). The local bladder serosa layer was cut with an electric knife and after the nodule was completely exposed, a bone biting forceps was used to snip it from the basin wall of the pubic symphysis. The rough surface of the nodule was polished with a bone file until it was flush with the basin wall and then sealed with bone wax. No damage was found in the muscular layer of the bladder. The cut tissue was sent for pathological examination (Fig. 4). The discomfort symptoms of the patient such as frequent and painful micturition were significantly relieved after the operation. The international prostate symptom score (IPSS) score was used as the template to evaluate the symptoms of frequent, painful, and laborious micturition [2]. Before the operation the patient’s score was 34 points (i.e., severe symptoms) and 4 days after the operation was 12 points (i.e., moderate symptoms).

## Discussion and conclusion

Reference to relevant literature published in the last 10 years [3–8] showed more than a dozen cases of lower urinary tract symptoms caused by pubic symphysis degeneration, including 15 cases of a pubic symphysis cartilage cyst, 1 case of a pubic symphysis osteomyelitis abscess, and 1 case of pubic symphysis osteochondritis. These cases were mainly postmenopausal women, some with a history of delivery trauma and perineal trauma. The main clinical symptoms were frequent urination, pain in the retropubic region, urinary incontinence, and other lower urinary tract symptoms. The majority of these cases were diagnosed by pelvic CT or MRI, with the symptoms improving significantly after surgery. According to the literature, osteochondritis of the pubic symphysis is a self-limited disease, which can be relieved by conservative treatment such as drugs and physiotherapy, but may easily reoccur. However, surgical intervention is needed if the condition is complicated by infection and degenerative changes such as bone destruction and hyperosteogeny [9].

Interestingly, another case of bladder pseudotumor caused by late degeneration of the pubic symphysis has been reported that was very similar to the present case [4]. In this case, the patient was treated for symptoms of a lower urinary tract infection. Bladder color ultrasound and cystoscopy showed a lump on the anterior wall of the bladder, although the surface mucosa was normal. After transurethral resection of the bladder tumor, pathology showed that the necrotic mass was full of fibrin and had no tumor cell characteristics. However, a mass was found again at the same location 3 weeks after the operation. Further pelvic MRI examination showed progressive degenerative changes of pubic symphysis, with connective tissue formed in the anterior wall of the bladder. During the course of treatment in our patient we also considered the possibility of bladder wall tumors, and if we had not performed a pelvic CT and MRI we may have encountered the same problem. Therefore, pelvic CT and nuclear magnetic resonance examination are necessary for diagnosis and appropriate treatment of unexplained lower urinary tract symptoms, especially in patients with bladder compression.

A previous study reported that about 55.5% of Chinese adult women have at least one lower urinary tract symptom, with an increase in the number of vaginal deliveries and deliveries confirmed to be risk factors for these symptoms [10]. Therefore, degeneration of the pubic symphysis caused by transvaginal delivery may be an important cause of female lower urinary tract symptoms.

Pathology suggested that urate deposition may have been a possible pathogenic factor in our patient despite her uric acid level being normal. Relevant literature reports that urate deposition is related closely to hyperuricemia and occurs rarely in patients with a normal serum uric acid level. Hyperuricemia often involves distal hand and foot soft tissue and joints and although it is common for uric acid crystals to invade cartilage, this is rare in the pubic symphysis [11, 12]. Due to a lack of relevant literature, there is no further discussion on hyperuricemia in this paper (Additional file 1).

In conclusion, female lower urinary tract symptoms are often encountered by urologists and the present case provides relevant experience on the diagnosis and treatment of female lower urinary tract symptoms (Additional file 2).

## Supplementary Information

**Additional file 1.** CARE checklist.

**Additional file 2.** Pathological report.

## Data Availability

Data sharing is not applicable to this article as no datasets were generated or analyzed during the current study.

## Abbreviations

CT: Computed tomography
MRI: Magic resonance imaging
PTH: Parathyroid hormone
IPSS: International prostate symptom score
