# Supplementary material for: Lower urinary tract symptoms in an elderly women caused by degeneration of the pubic symphysis
Source: BMC Urol. 2022 Jul 6;22:98. doi: 10.1186/s12894-022-01052-1 (PMC9261076; doi:10.1186/s12894-022-01052-1)
Supplement: Supplementary file 1 — Additional file 1. CARE checklist. [file 12894_2022_1052_MOESM1_ESM.pdf]

CARE Checklist of information to include when writing a case report

CARE case report guidelines

(cc) BY-NC-ND

Topic	Item	Checklist item description	Reported on Line
**Title**	1	The diagnosis or intervention of primary focus followed by the words "case report"	Title Page 1, line 2
**Key Words**	2	2 to 5 key words that identify diagnoses or interventions in this case report, including "case report"	Keywords Page 2, line 21
**Abstract (no references)**	3a	Introduction: What is unique about this case and what does it add to the scientific literature?	Page 2,conclusion section
	3b	Main symptoms and/or important clinical findings	Page 2, Case presentation
	3c	The main diagnoses, therapeutic interventions, and outcomes	Page 2, Case presentation
	3d	Conclusion—What is the main "take-away" lesson(s) from this case?	Page 2, Conclusions
**Introduction**	4	One or two paragraphs summarizing why this case is unique (**may include references**)	Page 3, Case presentation
**Patient Information**	5a	De-identified patient specific information.	Page 3, line 11
	5b	Primary concerns and symptoms of the patient.	Page 3, Case presentation
	5c	Medical, family, and psycho-social history including relevant genetic information	N/A
	5d	Relevant past interventions with outcomes	Page 3, line 16 to 21
**Clinical Findings**	6	Describe significant physical examination (PE) and important clinical findings.	Page 4, line 1 to 5
**Timeline**	7	Historical and current information from this episode of care organized as a timeline	N/A
**Diagnostic Assessment**	8a	Diagnostic testing (such as PE, laboratory testing, imaging, surveys).	Page 4, line 1 to 5
	8b	Diagnostic challenges (such as access to testing, financial, or cultural)	N/A
	8c	Diagnosis (including other diagnoses considered)	Page 4, line 11
	8d	Prognosis (such as staging in oncology) where applicable	Page 4, line 22
**Therapeutic Intervention**	9a	Types of therapeutic intervention (such as pharmacologic, surgical, preventive, self-care)	surgical
	9b	Administration of therapeutic intervention (such as dosage, strength, duration)	N/A
	9c	Changes in therapeutic intervention (with rationale)	Page 5, line 1 to 3
**Follow-up and Outcomes**	10a	Clinician and patient-assessed outcomes (if available)	Page 5, line 1 to 3
	10b	Important follow-up diagnostic and other test results	N/A
	10c	Intervention adherence and tolerability (How was this assessed?)	N/A
	10d	Adverse and unanticipated events	N/A
**Discussion**	11a	A scientific discussion of the strengths AND limitations associated with this case report	N/A
	11b	Discussion of the relevant medical literature **with references**.	Page 5, paragraph 2
	11c	The scientific rationale for any conclusions (including assessment of possible causes)	Page 5 to 6,Discussion and conclusion
	11d	The primary "take-away" lessons of this case report (without references) in a one paragraph conclusion	Page 6, paragraph 4
**Patient Perspective**	12	The patient should share their perspective in one to two paragraphs on the treatment(s) they received	Page 4, paragraph 2
**Informed Consent**	13	Did the patient give informed consent? Please provide if requested	**Yes** ☑ **No** ☐
