# Supplementary material for: Lower urinary tract symptoms in an elderly women caused by degeneration of the pubic symphysis
Source: BMC Urol. 2022 Jul 6;22:98. doi: 10.1186/s12894-022-01052-1 (PMC9261076; doi:10.1186/s12894-022-01052-1)
Supplement: Supplementary file 2 — Additional file 2. Pathological report. [file 12894_2022_1052_MOESM2_ESM.pdf]

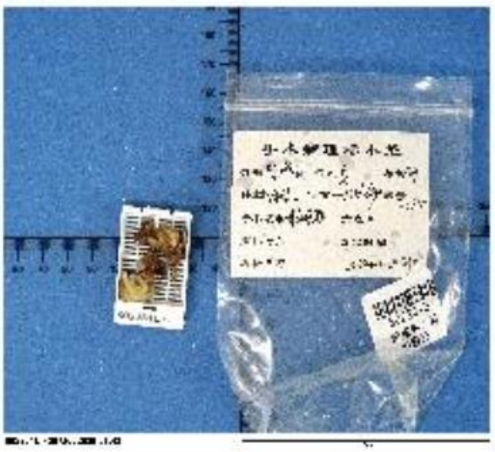

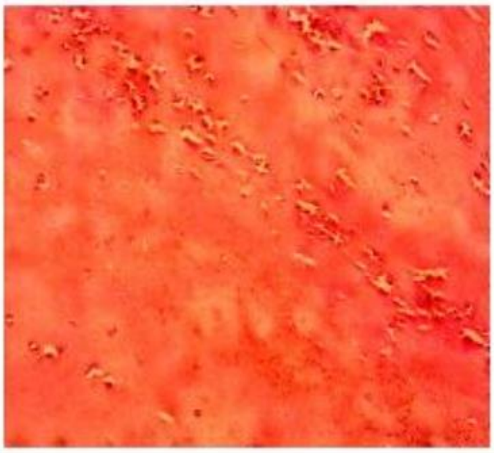

Pathological diagnosis:

【Pelvis】The degenerative cartilage tissue was examined, and the radial fine needle-like crystals destroyed the cartilage tissue and formed local calcification. It is considered to be caused by urate deposition.

(note: As reported in the literature, urate deposition is closely related to hyperuricemia, and can also occur in patients with normal serum uric acid and hypertensive cardiovascular diseases, often involving distal hand, foot, soft tissue and joints, although it is easy to invade cartilage, but it is rare to occur in the pubic symphysis. Please differentiate it from idiopathic articular osteomalacia (pseudogout) in combination with other relevant examinations. Further external consultation if necessary.)
